# Ischemic stroke as an initial presentation of neurosyphilis in a newly diagnosed HIV patient: A case report and literature review

**DOI:** 10.1002/ccr3.8794

**Published:** 2024-05-10

**Authors:** Moaz O. Moursi, Wael Hamam, Adnan Hajjar, Mohammad Es‐Salim, Soha Aboukhalaf, Omar Jamil, Muhammad Zahid

**Affiliations:** ^1^ Department of Internal Medicine Hamad General Hospital Doha Qatar; ^2^ College of Medicine, QU Health Qatar University Doha Qatar; ^3^ Department of Radiology Hamad General Hospital Doha Qatar; ^4^ Weill Cornell Medical College Doha Qatar

**Keywords:** case report, HIV, ischemic stroke, neurosyphilis, syphilis

## Abstract

With syphilis resurgence, physicians should be more vigilant to infection‐induced cerebral vasculitis in high‐risk patients presenting with neurological symptoms. In this case, neurosyphilis should not be missed. Thorough serologic screening and lumbar puncture are crucial for diagnosis, and further research is needed for safe and effective treatments in these populations.

## 1 INTRODUCTION

Stroke disease remains a leading cause of morbidity and mortality worldwide. Strokes can affect all ages, with approximately 10%–15% of all strokes occurring in young adults aged 18–50 years of age, and recent trends showed an increased rate of strokes among young adults.^1^ Among young individuals, the most prevalent cause of ischemic strokes is arteriopathies, primarily nonatherosclerotic ones such as dissection, along with premature atherosclerosis.^2^ One of the important differentials to consider is neurosyphilis (NS), especially in high‐risk populations.^3^

Syphilis is a prevalent sexually transmitted disease caused by the bacterium *Treponema pallidum*.^4^ The primary risk factors associated with contracting this disease include illicit drug usage, such as cocaine and methamphetamine, as well as engaging in sexual activities among men who have sex with men.^5^

Syphilis can impact the nervous system in diverse ways, causing neuropsychiatric symptoms, myelopathy, seizures, strokes, ocular manifestations, and brain stem or cranial nerve abnormalities.^6^ In cases of cryptogenic stroke in young patients, it is important to consider NS as a possible cause, given that about 15% of untreated NS patients and nearly 3% of all syphilis patients present with a stroke, particularly those under 50 years of age.^7^

## 2 CASE HISTORY/EXAMINATION

A 36‐year‐old gentleman of Indian origin presented to the emergency department at 2:30 a.m. with right upper and lower limb weakness associated with three episodes of vomiting. He woke up around 12 a.m. with headache and vomiting. He then noticed numbness and weakness in the right side of the body. He denied any visual or speech problems. He had gone to bed in his usual state of health around 9 p.m. In the last 2 weeks, he complained of odd sensations and occasional tremors in the right arm and leg, for which he had not sought medical attention.

He had no significant past medical history and did not take any medications regularly. He worked as a teacher. He was a nonsmoker without any history of alcohol or drug abuse.

On examination, he had a regular pulse of 88/min, blood pressure of 124/87 mmHg, and oxygen saturation of 98% on room air. His Glasgow Coma Scale was 15/15, and he had normal speech. Partial ptosis of the left eye and bilateral torsional nystagmus were noted in both vertical and horizontal gaze movements. Both pupils were equal and reactive to light, and no external ophthalmoplegia was noted. The tone was normal in all four limbs; power was 0/5 in the right upper limb proximal and distally, while it was 3/5 proximally and 2/5 distally in the right lower limb. Power on the left side was normal. Reflexes were exaggerated on both sides, with ill‐sustained ankle clonus noted on the right side. The right plantar was upgoing, while it was down‐going on the left side. Fine and crude touch, pinprick, and joint position sensations were intact bilaterally. His National Institute Health Stroke Scale score (NIHSS) was 7, indicating a moderate stroke (stroke range 0–42, with increasing values indicating increasing severity). Respiratory and cardiovascular systems were normal on examination.

## 3 METHODS (INVESTIGATIONS, DIFFERENTIAL DIAGNOSIS, AND TREATMENT)

**FIGURE 1:**
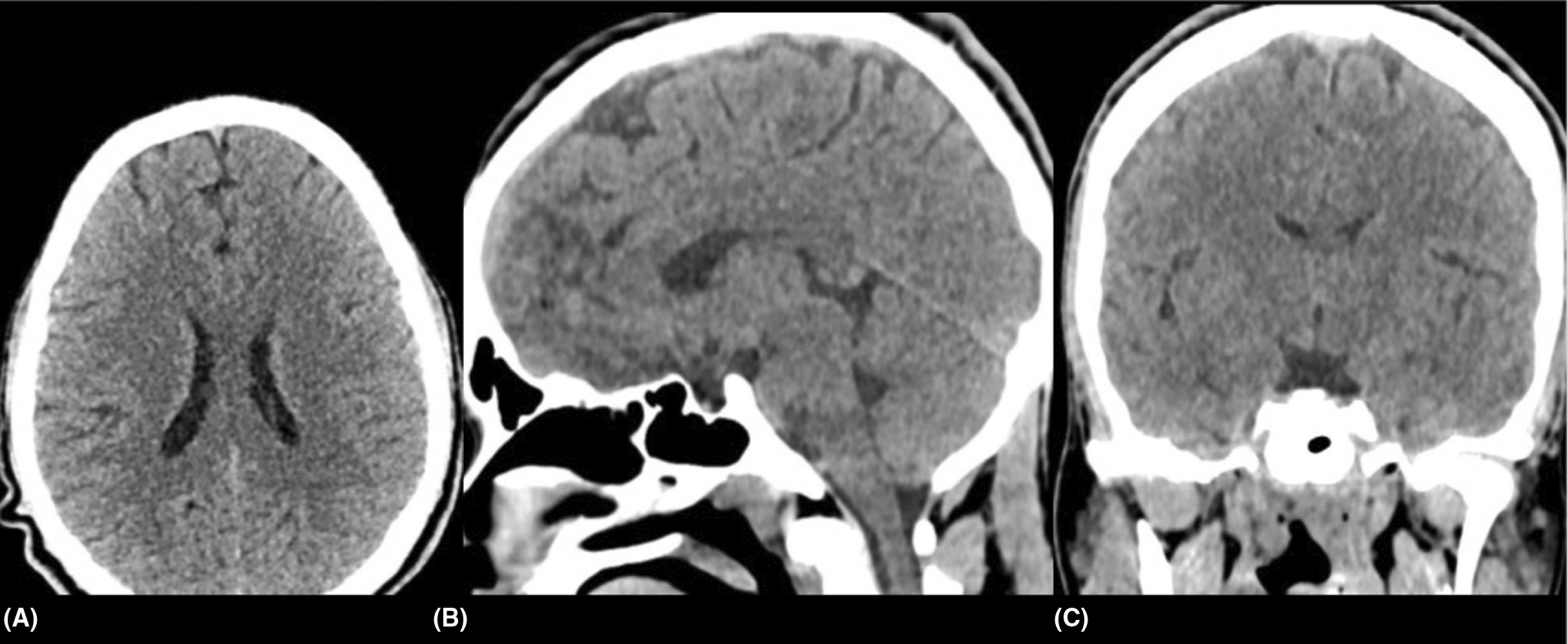
Non‐contrast CT head: (A) axial, (B) sagittal and (C) coronal cuts, showed no acute pathology.

**FIGURE 2:**
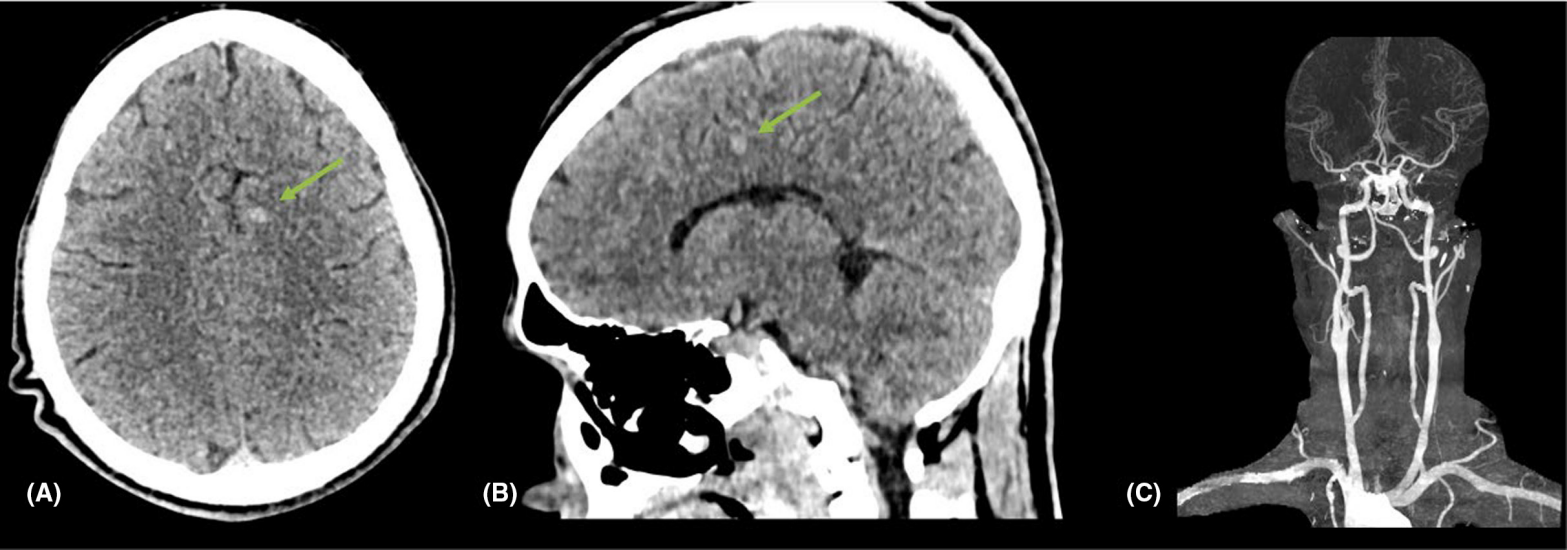
Non‐contrast CT head: (A) Axial and (B) Sagittal selected cuts, showed a hyperdense focus measuring 4 × 6 mm devoid of perilesional oedema in the left parasagittal frontal lobe region (green arrows) suggestive of a cavernoma or a petechial hemorrhage. (C) A CT intracranial angiogram was unremarkable.

A non‐contrast CT scan brain study showed no acute pathology (Figure 1); however, an incidental finding of a small cavernoma in the left frontal parasagittal cortical area was noted (Figure 2A, B). CT angiography showed normal opacification of cervical and intracranial segments of major arteries, and CT perfusion study maps appear fairly symmetric to the visualized images (Figure 2C). Given the history and examination, time of onset of symptoms, and CT brain findings, the Stroke team decided not to proceed with any intervention. The patient was admitted for further workup.

The complete blood picture, clotting profile, thyroid, kidney, and liver functions were normal except for raised total proteins of 87 gm/L (Normal 60–80 gm/L). A chest x‐ray, 12‐lead electrocardiogram, and transthoracic echocardiography, including bubble study, were reported as normal. His HbA1c was recorded at 5.4%. His total cholesterol was 3.6 mmol/L (desirable <5.2), triglycerides 0.9 (desirable <1.7), high‐density lipoproteins 0.7 mmol/L (desirable 1), and low‐density lipoproteins 2.5 (optimal <2.59).

**FIGURE 3:**
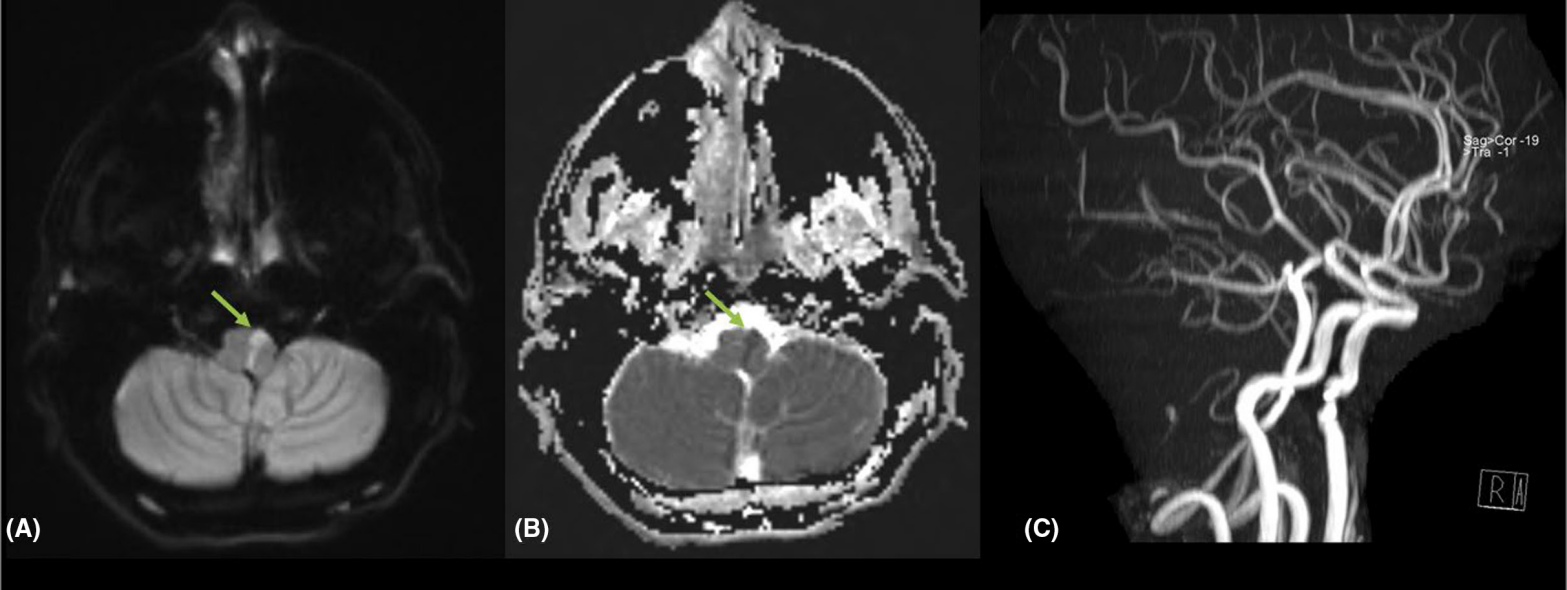
MRI head axial cuts: (A) DWI and (B) ADC, showed a linear area of diffusion restriction along the left paramedian aspect of the upper medulla oblongata with high signal intensity in diffusion‐weighted images (DWI) and low value in ADC map (green arrows) denoting recent ischemic infarction. There was no evidence of hemorrhagic components within. (C) Intracranial MRA was unremarkable with no major vascular occlusion, stenosis, aneurysm, or AVM.

**FIGURE 4:**
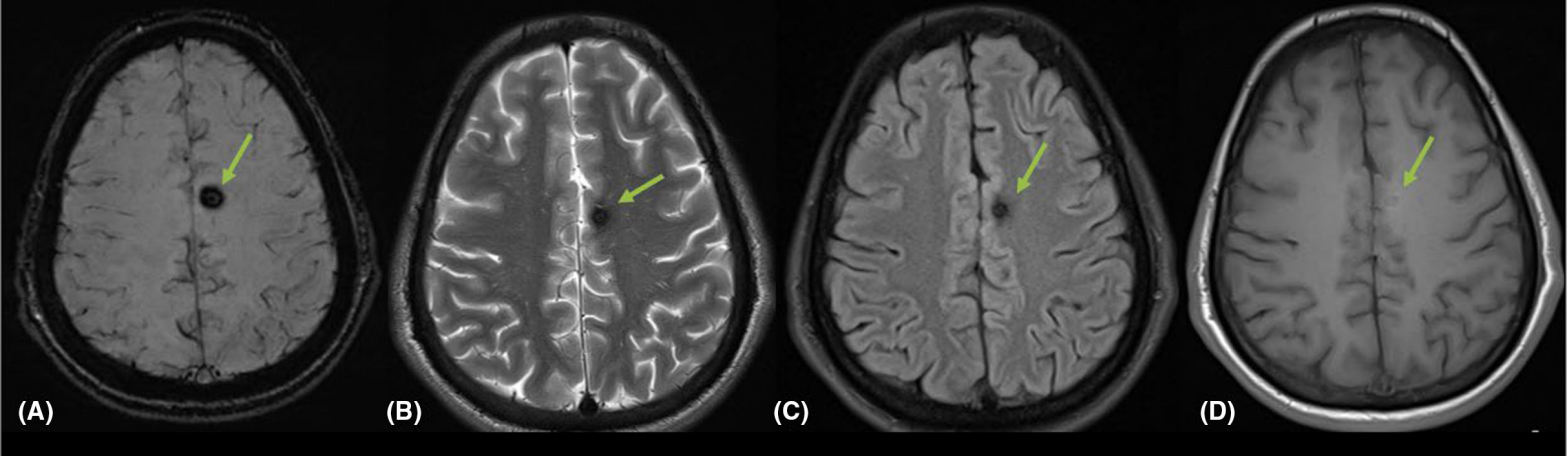
MRI head selected axial cuts: (A) SWI, (B) T2, (C) FLAIR and (D) T1, showed a focal area of significant SWI blooming measuring 5.5 mm in the left frontal parasagittal area (green arrows) that showed significant low T2, FLAIR, T1 signal intensity; features suggestive of cavernoma.

An MRI scan of the brain showed a small recent ischemic infarction along the left paramedian aspect of the medulla oblongata with no hemorrhagic transformation (Figure 3A, B); the incidental small cavernoma was noted as well (Figure 4). The MR angiography of the brain revealed no significant intracranial arterial abnormality (Figure 3C).

A diagnosis of ischemic stroke in a 36‐year‐old gentleman with no apparent risk factors was made, and further investigations were initiated to look for the cause. Autoimmune profile, including Antinuclear antibody, Antiphospholipid screen, and Antineutrophilic cytoplasmic antibody, returned negative. A 48‐h Holter monitoring did not show any tachy or brady arrhythmias. A limited thrombophilia screen also revealed no abnormalities.

Syphilis serology results were positive: reactive rapid plasma regains (RPR) with a titre of 1:64 and reactive *T pallidum* particle agglutination (TPPA) assay. Hepatitis B and C serology were negative; however, HIV serology came back positive. CD13 count was normal, but the CD3^+^/CD4^+^ percentage was low at 6.20% (Normal 31.45%–62.38%). CD3^+^/CD8^+^ T cells and Natural killer cells were increased, while CD4^+^ T cells were decreased.

Cerebrospinal fluid (CSF) examination showed an increased white blood cell count of 427 with 98% lymphocytes, 1% neutrophils, and monocytes each, and two red blood cells. CSF albumin was raised at 1145 mg/L (normal 0–350), CSF lactic acid 3.4 mmol/L (normal 1.1–2.4), CSF IgG 667 mg/L (normal 0–34), and CSF IgG index 1.1 (normal 0.3–0.6). CSF VDRL also returned as reactive.

CSF Viral PCR screen was negative, including human simplex virus (HSV 1&2), Human Herpes virus (HHV6), and Cytomegalovirus. CSF was also negative for cryptococcal antigen, tuberculosis (TB) PCR, and TB and fungal culture. Urine testing for *chlamydia trachomatis* and *Neisseria gonorrhoeae* DNA were negative.

A diagnosis of acute ischemic stroke secondary to NS with concurrent HIV infection was made. The infectious disease team started on intravenous benzylpenicillin 4 million units four hourly for a total of 14 days and started antiretroviral treatment for the HIV infection. The stroke team advised to continue with aspirin only for secondary prevention.

## 4 CONCLUSION AND RESULTS (OUTCOME AND FOLLOW‐UP)

The patient was transferred to a rehabilitation facility and continued to improve with active physiotherapy. He recovered well, and his modified Rankin's score was two upon discharge. However, he did not show up to follow up later, suggesting that he may have returned to his home country.

## 5 DISCUSSION

This is a case of a 36‐year‐old gentleman with NS with concurrent HIV infection and no other risk factors presented with ischemic stroke likely secondary to meningovascular syphilis. Syphilis is a bacterial disease caused by *T pallidum*, which, if undiagnosed, can affect all organ systems. In UK, syphilis incidence rates have been on the rise, increasing from 6.2 per 100,000 population in 2013 to 15.4 per 100,000 population in 2022 (148% rise).^8^

NS, a *T pallidum* infection of the central nervous system (CNS), develops in about 30% of untreated syphilis patients, and recent studies reported symptomatic NS between 3% and13% among the infected patients.^9^, ^10^ NS usually presents 5–7 years from the time of initial syphilis infection^11^ and can be divided into two groups. Early NS can be asymptomatic or present with acute syphilitic meningitis, gumma, or stroke as a result of meningovasculitis, while late presentation includes tabes dorsalis and neuropsychiatric symptoms.^12^, ^13^

Meningovascular syphilis causes endarteritis (Huebner arteritis) of all size vessels, characterized by lymphocytic infiltration of adventitia, thinning of media, and fibroblast proliferation of intima, which leads to occlusion of arteries, causing ischemic stroke. Nearly 15% of untreated NS patients present with ischemic stroke, as was the case of our patient.^14^

NS is a great imitator; its presentation varies and is confused with many different disorders, which is further complicated because there is no gold standard test to diagnose NS. CSF studies (CSF cell counts, protein, and CSF—VDRL) are required to diagnose NS in patients with reactive serological tests and neurological signs and symptoms. Elevated proteins and white blood cells in CSF are found in NS.^15^ Moreover, diagnosis requires a positive CSF VDRL test, which has high specificity but low sensitivity and can be false‐negative in up to 70% of the late syphilis cases.^16^

In a systematic review of 149 case reports and 93 retrospective case series, the serological treponemal test positivity was 95% compared to a 75% non‐treponemal test positivity rate, and elevated CSF WBCs and proteins were observed in about 70% of the NS patients.^17^ Studies reported the specificity of the CSF‐TPPA test as 49%–84%, and guidelines vary in recommending this test for NS diagnosis.^18^, ^19^ CSF study in our patient showed raised WBCs (427 cells), raised proteins, and reactive CSF‐VDRL, favoring a diagnosis of NS.

The Centre for Disease Control and Prevention (CDC) recommends a lumbar puncture in syphilis patients if serum RPR titre ≥1:32, as they are most likely to have CSF changes consistent with NS.^20^ The prevalence of syphilis in HIV‐infected people is much higher than in the general population.^21^ CDC also recommends a cut‐off CSF WBC of greater than 20 for NS diagnosis in patients with concurrent HIV infection, as HIV infection itself causes CSF pleocytosis.^18^ CDC recommends intravenously 3–4 million Penicillin G every 4 h for 10–14 days for NS treatment.^18^ Our patient had a serology RPR titre of 1:64 and CSF WBCs of 427 with 98% lymphocytes with reactive CSF VDRL, making NS a likely diagnosis. He was treated with the same regimen as recommended by the CDC.

Neuroimaging findings in NS include cortical or subcortical infarcts; the radiological characteristics on MRI scans are not highly specific but very sensitive in detecting changes of cerebral vasculitis, usually including medium and small vessels. Two‐thirds of the neuroimaging in NS patients was reported to be normal or nonspecific brain atrophy secondary to chronic meningoencephalitis.^5^, ^14^ Other changes on the MRI scan, including high signal changes in the bilateral temporal lobes, leptomeningeal granulomas, meningeal enhancement, and arteritis, were documented.^22^ MRI scan of our patient was reported as normal, apart from a recent infarction in the posterior circulation territory and an incidental finding of cavernoma.

HIV infection predisposes individuals to neurological pathologies. It increases the risk of ischemic stroke, especially with a CD4 cell count of less than 350 × 106 cells/L, either due to HIV‐induced vasculopathy or opportunistic co‐infection‐related arteritis with organisms like varicella‐zoster virus, syphilis, and tuberculosis, etc. The safety of thrombolytic therapy for acute ischemic stroke in HIV patients is not well established. Although thrombolytic therapy has been used successfully for myocardial infarction in HIV‐infected patients, it could be potentially harmful to patients with vasculitis secondary to NS and or HIV infection due to increased bleeding risk from fragile vessel walls.^23^ Long‐term antiplatelet agents are indicated for prevention, especially if other stroke risk factors exist.^24^ With these factors in mind, the stroke team chose to start Aspirin as secondary prevention for our patient.

In conclusion, the resurgence of syphilis, particularly in those with HIV, highlights the significance of exploring CNS infections as potential culprits behind neurological deficits, especially among stroke patients with no apparent risk factors or identified cause. NS can manifest with a wide spectrum of neurological symptoms, and acute stroke can be the primary symptom, especially in untreated patients. For cryptogenic stroke patients, particularly in high‐risk groups, thorough serologic screening for NS and HIV is crucial and should be followed by lumbar puncture to confirm CNS involvement. There is no gold standard test to diagnose NS; reactive CSF VDRL and TPPA tests highly suggest NS, but a negative test doesn't rule out NS. The safety and efficacy of intravenous thrombolysis in NS or HIV‐infected individuals in acute ischemic stroke settings require further research, as the incidence of stroke in this population may rise with increased life expectancy. Treatment for NS and HIV is standardized, but more research is needed for secondary stroke prevention measures in these groups of patients.

## AUTHOR CONTRIBUTIONS

**Moaz O. Moursi:** Conceptualization; data curation; methodology; project administration; writing – original draft; writing – review and editing. **Wael Hamam:** Data curation; writing – original draft. **Adnan Hajjar:** Writing – original draft. **Mohammad Es‐Salim:** Writing – original draft. **Soha Aboukhalaf:** Writing – review and editing. **Omar Jamil:** Writing – original draft. **Muhammad Zahid:** Conceptualization; methodology; project administration; supervision; writing – review and editing.

## FUNDING INFORMATION

This research did not receive any specific grant from any funding agencies.

## CONFLICT OF INTEREST STATEMENT

There is no conflict interest to be declared.

## ETHICS STATEMENT

All methods were performed in accordance with the relevant guidelines and regulations. Written informed consent was obtained from the patient.

## CONSENT

Written informed consent was obtained from the patient for publication of this case report and any accompanying images. A copy of the written consent is available for review by the Editor‐in‐Chief of this journal on request.

## Data Availability

Data sharing is not applicable to this article as no datasets were generated or analyzed during the current study.
